# Method for the Routine Determination of Accurate Masses by Triple Quadrupole Mass Spectrometry

**DOI:** 10.3390/mps1010009

**Published:** 2018-02-14

**Authors:** Pedro A. Segura, Killian Barry, Emmanuel Eysseric, Shawn Gallagher-Duval, Philippe Venne, Guillaume Bélanger

**Affiliations:** Department of Chemistry, Université de Sherbrooke, Sherbrooke, QC J1K 2R1, Canada; killian.barry@usherbrooke.ca (K.B.); Emmanuel.Eysseric@usherbrooke.ca (E.E.); Shawn.Gallagher-Duval@usherbrooke.ca (S.G.-D.); philippe.venne@usherbrooke.ca (P.V.); Guillaume.Belanger@usherbrooke.ca (G.B.)

**Keywords:** spectral accuracy, organic synthesis, mass spectrometry, low-resolution mass spectrometry, triple quadrupole, small organic molecules

## Abstract

A new method for the measurement of accurate masses using direct infusion in an electrospray-triple quadrupole mass spectrometer is presented and compared to the traditional method using high-resolution mass spectrometry. The proposed method uses internal calibrants and post-acquisition calibration of the mass spectrum signal using the MassWorks software to determine accurate masses. Then, based on parameters such as elemental composition, number of double bond equivalents, and type of ion (even- or odd-electron), etc., a list of potential molecular formula candidates are generated and ranked according to spectral accuracy, (i.e., similarity between the calibrated profile and theoretical isotopic patterns). Experiments using six diverse synthesis products showed that mass accuracy in the Quattro Premier triple quadrupole mass spectrometer (QqQMS) was ≤9.2 mDa and spectral accuracy was ≥90.6%. According to both mass accuracy tolerance (±10 mDa) and spectral accuracy, the correct molecular formula was ranked in the top seven compounds out of up to 32 potential candidates. When considering the context of the synthesis reaction, only one formula was possible. In summary, results showed that the measurement of spectral accuracy in a low-resolution instrument such as the triple quadrupole was strongly dependent on the signal intensity and the presence of interfering peaks in the profile mass range window. This study suggests that use of triple quadrupole mass spectrometry followed by post-acquisition calibration can be an economical and robust approach compared to the traditional method using high-resolution mass spectrometers for the measurement of accurate masses in routine applications using small organic molecules at microgram-per-litter concentrations in relatively clean matrices.

## 1. Introduction

The application of accurate mass measurements for the determination of molecular formulas of reaction products is a well-established technique in organic chemistry [1]. This technique was first proposed by Beynon [2], and is essential in the toolbox of routine techniques performed by modern organic chemists to verify the success of synthesis experiments. Such mass measurements are performed with high-resolution instruments like magnetic sectors or time-of-flight mass spectrometers that can measure masses with high accuracy, usually with a mass error lower than a few millidaltons. While high-resolution and accurate mass are often lumped together, they are distinct and separate concepts. It is possible to perform accurate mass analysis with a low-resolution mass spectrometer if adequately calibrated [3]. While high mass resolution is indispensable when working with complex matrices for adequate accuracy in mass determination, for the routine analysis of organic synthesis products, there are many advantages of using quadrupole mass spectrometers compared to high-resolution instruments, such as ease of use, robustness, and cost. In fact, there have been previous works using liquid chromatography coupled to quadrupole mass analyzers for the accurate mass measurement of small organic molecules. 

In 1996, a study on the measurement of accurate masses using a low-resolution single quadrupole mass spectrometer was introduced by Tyler, et al. [4]. They analyzed polar organic molecules with *m/z* ranging from 190 to 750, mean mass errors were all within 4.5 ppm (1.1 mDa) and standard deviations were less than 3 ppm. Results obtained in their study were comparable to what was achieved using high-resolution mass spectrometers. In a multi-laboratory intercomparison study on accurate mass measurement using multiple mass analyzers and ionization sources published in 2003 by Bristow and Webb [5], two laboratories produced <10 ppm (≈4.8 mDa) mass accuracy measurements for a small organic molecule using triple quadrupole mass spectrometers. In the same year, a method for metabolite identification using a unit mass resolution quadrupole-linear ion trap mass spectrometer was proposed in by Gu, et al. [6]. They used a software-based post-acquisition procedure to externally calibrate the instrument. This method corrects both mass axis and spectral peak shape to facilitate metabolite identification with high mass accuracy. Mass errors ranged from 1.1 to 11.1 mDa with their method. A paper by Grange, et al. [7] introduced the measurement of both mass accuracy and relative isotopic abundances of precursor and product ions in a liquid chromatograph-triple quadrupole mass spectrometer to elucidate the unique molecular formulas of model small-molecule compounds. While these techniques successfully showed that the determination of molecular formulas is possible using low-resolution mass spectrometers, their widespread application has been hampered by access to high-end triple quadrupole mass spectrometers capable of performing accurate mass measurements and lack of automatization, which make the elucidation process time consuming and cumbersome.

The objective of the present study was to apply a post-acquisition mass calibration procedure similar to the one developed by Gu et al. to determine the accurate mass of synthetized small organic molecules introduced by direct infusion into a triple quadrupole mass spectrometer. This procedure takes only a few minutes and could facilitate the routine use of mass spectrometry for the monitoring and optimization of organic synthesis experiments.

## 2. Material and Methods

### 2.1. Reagents and Chemicals

**Figure 1:**
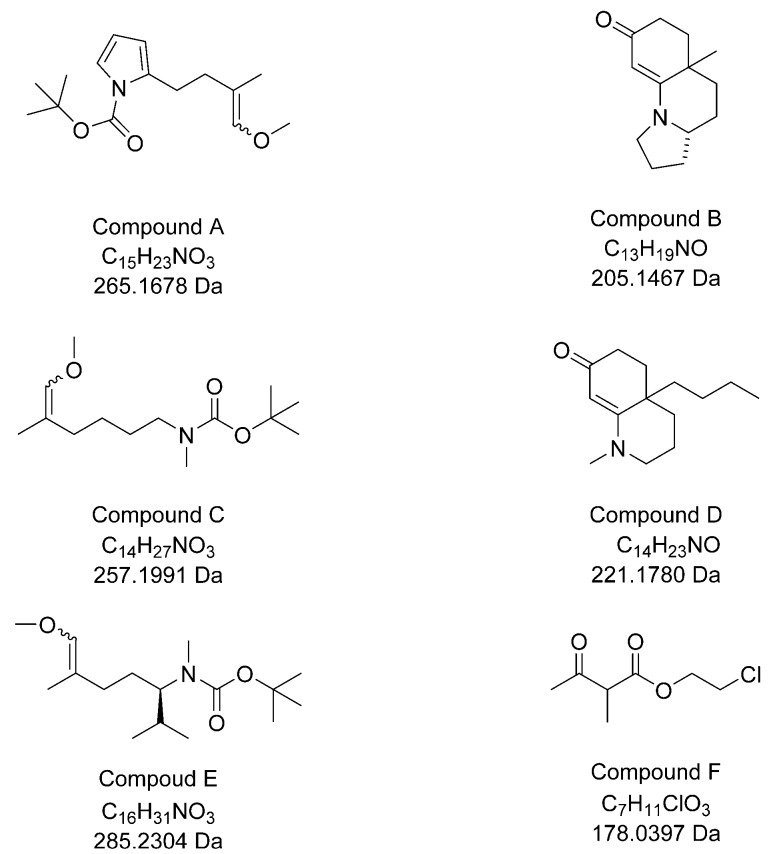
Molecular structures, formulas, and monoisotopic neutral masses of the synthetic compounds used in this study.

Six compounds synthetized in the organic chemistry laboratory of G. Bélanger (Université de Sherbrooke, QC, Canada) were used to test the application of the technique. These compounds (A–F, Figure 1) were either intermediates (A, C, E, and F) used in the preparation of certain polycyclic enaminones, or enaminones (B and D) [8]. The structures of these compounds were confirmed by regular characterization techniques such as infrared and proton and carbon-13 nuclear magnetic resonance.

### 2.2. Instruments

For high-resolution measurements, a maXis quadrupole-time-of-flight mass spectrometer (QqTOFMS) from Bruker (Billerica, MA, USA) was used. In this instrument, full width at half maximum resolution (*R_FWHM_*) for ion *m/z* 222.1853 was ≈17,000. External mass calibration, stable for a period of approximately 3 h, was performed using a solution of sodium formate. Then, each compound was dissolved in methanol and infused individually into the QqTOFMS using a syringe pump at a flow rate of 5 µL·min^−1^. Conditions of electrospray ionization in the positive mode (ESI+) were as follows: capillary voltage (1200 V), end plate offset (−500 V), nebulizer (0.4 bar), nitrogen dry gas (4.0 L·min^−1^), and dry gas temperature (180 °C). Mass analyzer parameters were as follows: range (*m/z* 50 to 1200), ion cooler radiofrequency (RF) voltage (45 Vpp), funnel RF voltage (250 Vpp). Transfer parameters were optimized to adjust the ion intensity to 10^5^ counts s^−1^. Spectra were acquired with a time resolution of 1 s.

For low-resolution measurements, a Quattro Premier triple quadrupole mass spectrometer (QqQMS) from Waters Corp. (Milford, MA, USA) was employed. Before analysis, external calibration of the QqQMS was done using a solution of sodium formate. In this instrument, *R_FWHM_* for ion *m/z* 222.2 was ≈500. Next, each compound was infused individually using a syringe pump at a flow rate of 20 μL·min^−1^. Conditions of electrospray ionization in the positive mode (ESI+) were the following: capillary voltage (2500 V), cone voltage (20 V), cone gas flow (50 L·h^−1^), desolvation gas flow (50 L·h^−1^), desolvation temperature (200 °C), source temperature (120 °C). Mass analyzer parameters were as follows: cycle time (0.6 s), scan duration (0.5 s), scan range (*m/z* 150–350). Mixtures of up to four internal mass calibrants were used for post-acquisition calibration depending on the analyte, and contained the following compounds: caffeine (monoisotopic mass: 194.0804 Da), atrazine (215.0938 Da), cyclophosphamide (260.0248 Da), metoprolol (267.1834 Da), avobenzone (310.1569 Da), and benzylbutyl phthalate (312.1362 Da). These internal calibrants were detected as protonated molecules [M+H]^+^ or sodium adducts [M+Na]^+^. All samples were infused once, and typically ≈30 acquisitions per sample were recorded for QqTOFMS measurements and ≈100 acquisitions per sample for QqQMS.

### 2.3. Post-Acquisition Mass Calibration and Determination of Molecular Formulas from Accurate Masses

The determination of molecular formulas from accurate masses from QqTOFMS data was done using the DataAnalysis software (version 4.2) of the instrument. The following parameters were applied: allowed elements and number of atoms (C_2–25_, H_0–50_, N_0–10_, O_0–10_, Na_0–1_, Cl_0–5_), mass tolerance (±10 mDa), charge (+1), rings plus double bonds (−0.5 to 20), and electron configuration (even). Only these elements were selected since others were not present in the initial reagents, or in the case of Na it is a ubiquitous contaminant in the mobile phases and causes the formation of sodium adduct ions that are detected by the mass spectrometer.

MassWorks version 4.0, developed by Cerno Bioscience (Norwalk, CT, USA), was used for post-acquisition calibration and determination of molecular formulas from accurate masses obtained at low resolution. In MassWorks, low-resolution mass spectra must first be calibrated in order to assign formulas to accurate masses. The software uses the experimental profile mass spectrum of external or internal standards to calibrate the line shape of the mass spectrometer to a known mathematical function. More details are given in the work of Wang et al. [9]. This calibration procedure takes only a few minutes, since it is a semi-automatic function of the software. Then, the calibrated data was processed using the calibrated line shape isotope profile search (CLIPS) algorithm. CLIPS first uses classic criteria such as mass tolerance, type and number of allowed elements, charge, and electron configuration to find the most likely molecular formula candidates. Then, the calibrated isotopic pattern of the unknown is compared to the isotopic pattern of those candidates, and a parameter called spectral accuracy (SA) is calculated for each potential candidate, based on the root mean square error between both isotopic patterns [3]. A perfect fit between calibrated and theoretical isotopic patterns means 100% SA. Therefore, the most likely candidates for a given accurate mass and its corresponding isotopic pattern are ranked according to SA. 

For the sake of comparison, common parameters used for accurate mass and SA determination of all compounds tested were the same as those used for the QqTOFMS data. For the CLIPS function, profile mass range start and end were set to −0.5 and 3.5 Da, respectively. This parameter indicates the width of the window of the profile mass spectrum relative to the monoisotopic peak. Using that setting, the M, M+1, M+2, and M+3 isotopic peaks are included in the calculation of SA. 

## 3. Results and Discussion

The result of the measurements of accurate masses in both QqQMS and QqTOFMS systems for the six synthetic compounds are shown in Table 1. As expected, ∆m with the QqTOFMS system was ≤2.6 mDa and superior to those obtained with the QqQMS after post-acquisition mass calibration (≤9.2 mDa). The largest observed difference in absolute terms of ∆m between the two methods was 9 mDa, for compound A. In the case of compound B, ∆m observed in the QqQMS (−0.1 mDa) was slightly better than the one observed in QqTOFMS (0.3 mDa). QqTOFMS results could have been even better using internal mass calibration. Nevertheless, it is remarkable that the present system used had more than 12 years of service and was not designed for accurate mass measurement, yet could obtain comparable results to those of a QqTOFMS. The impact of the post-acquisition calibration is illustrated with the data shown in Table 1. In only one case (compound A), the post-acquisition calibration resulted in a worse mass accuracy than the original experimental value. For the remaining compounds, mass accuracy improved up to 376 mDa. These results demonstrate that MassWorks software is able to correct large deviations in mass accuracy by using three-to-four internal calibrants.

Ranking of the molecular formulas according to SA showed that the correct formula was always among the top seven candidates (Tables S1–S6, Supplementary material). We did not include spectra accuracy measurements for QqTOFMS data in Table 1, since our objective was to compare a QqQMS-based method to a standard method of determining accurate mass based on high-resolution mass spectrometry. A previous study showed that SA in the QqTOFMS at high concentrations in pure solvents are very high, usually 97% or better [10]. A detailed examination of reaction reagents, as well as expected reaction products for each compound, demonstrated that the other candidate formulas were unlikely because the number of elements did not correspond (e.g., higher or lower number of O, N, or Cl than those in the employed reagents). For example, for compound E, the correct formula (C_16_H_31_NONa^+^) is ranked lower than six other formulas (C_13_H_30_N_3_O_5_^+^, C_12_H_27_N_7_ONa^+^, C_12_H_30_N_5_O_4_^+^, C_11_H_27_N_9_Na^+^, C_15_H_31_N_3_O_2_Na^+^, and C_13_H_26_N_9_^+^), but none of those are possible in the context of the synthesis of compound E. 

These results demonstrate that the measurement of accurate masses in a QqQMS using a post-acquisition mass calibration method is an interesting possibility for laboratories performing routine analyses of purified samples. An example of this would be the monitoring of synthesis experiments that are indispensable to confirm that specific reactions and purification steps were successful. While inter-day reproducibility could not be evaluated due to insufficient material being available, previous experiments [10] have shown that this method is reproducible with an average standard deviation of three replicate accurate mass measurements performed within the same day for four different small molecule compounds (nominal masses between 215 and 837 Da) of 0.5 mDa.

The improved ∆m of the QqQMS system is possible thanks to the data calibration performed by the MassWorks software. While the application of internal calibrants used in the QqQMS system was necessary to improve mass accuracy due to stability issues with the mass spectrometer [10], previous reports using single quadrupole mass spectrometers indicate that a similar level of performance could be also be achieved by using external calibrants [11,12,13] (that is, calibrants not added to the sample). Thus, calibration is a key step in the correct determination of accurate masses in MassWorks. During the experiments, it was observed that the choice of an improper profile mass window for the internal calibrants or the use of internal calibrants with low signals in the mass spectra resulted in important mass shifts (in some cases >10 mDa) in the calibrated experimental mass spectra, as well as a loss of SA.

**Figure 2:**
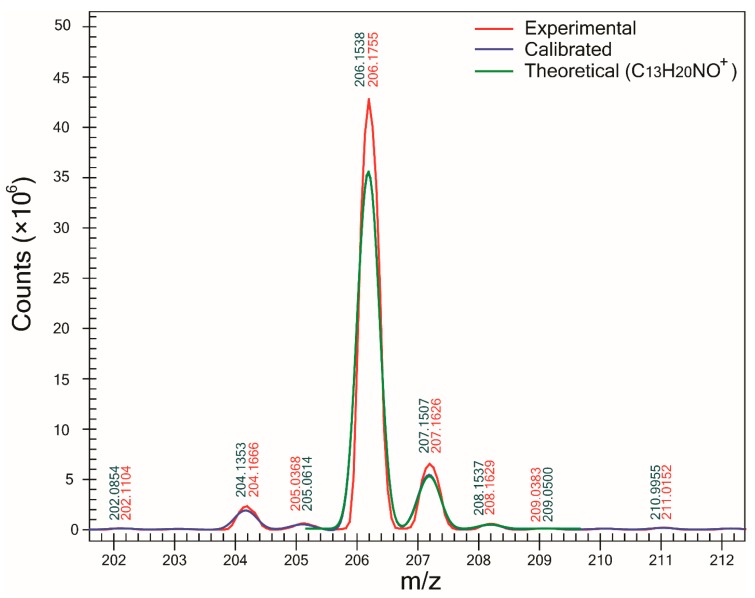
Experimental (red line) mass spectrum of the protonated molecule of compound B (C_13_H_20_NO^+^) acquired by QqQMS. The calibrated mass spectrum (blue line) was determined by MassWorks based on the post-acquisition internal calibration. Theoretical isotopic pattern (green line) of that protonated molecule was calculated by the software. SA was 98.9% and ∆m was −0.1 mDa. Numbers in red and blue correspond to centroid values of experimental and calibrated mass spectra, respectively.

**Figure 3:**
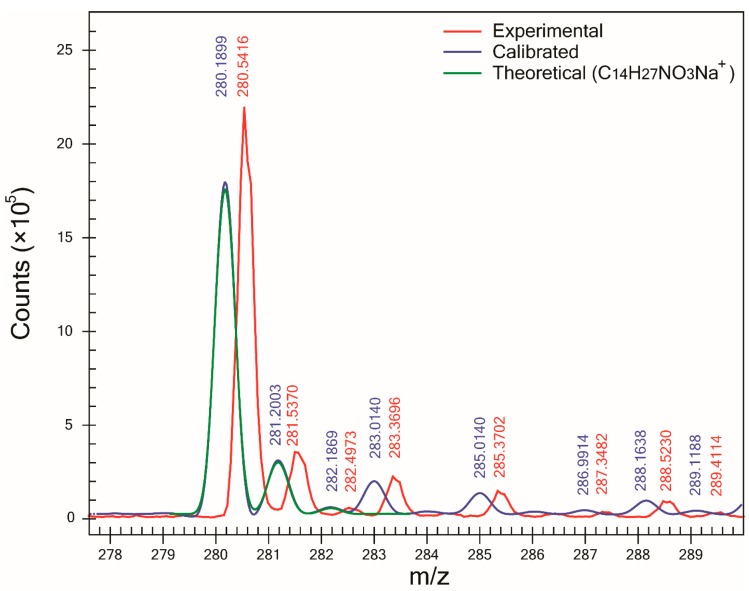
Experimental and calibrated mass spectra and theoretical isotopic pattern of the sodium adduct ion of compound C (C_14_H_27_NO_3_Na^+^). SA was 90.6% and ∆m was 1.6 mDa. Numbers in red and blue correspond to centroid values of experimental and calibrated mass spectra, respectively. Loss of SA was caused by the abundant peak at *m/z* 283.0140 that does not match the expected intensity of the M+3 of the theoretical isotopic pattern.

One of the major benefits of using the proposed method is the determination of SA which can be used as a complementary tool to ∆m in the assignment of molecular formulas to unknowns. For all compounds except one, SA was >93%, and in the case of compounds B (Figure 2) and F, SA was >98%. However, for compound C, the lowest SA (90.6%) was obtained. Examination of its mass spectra showed that compound C—which was detected as a sodium adduct at *m/z* 280.1899 (Figure 3)—was affected by the presence of an interference peak at *m/z* 283.0140, thus increasing the abundance of its M+3 peak. Since the abundance of the M+3 peak does not correspond to the expected value for an ion of molecular formula C_14_H_27_NO_3_Na^+^, the SA decreased. After modifying the profile mass range value in the CLIPS function from 3.5 to 2.5 Da (and leaving all other parameters equal), an increase in SA to 97.5% and ranking (first out of 14 potential candidates) was observed. That increase in both SA and ranking was the result of the exclusion of the peak at *m/z* 283.0097 in the SA calculation. It is interesting to point out that for compound F, the presence of a Cl atom yielding an abundant M+2 peak did not automatically result in high spectral accuracy and the highest ranking. In fact, the correct compound was ranked third since other two compounds (C_5_H_6_N_6_OCl^+^ and C_4_H_6_N_8_Cl^+^) had slightly better spectral accuracies, with less than 0.1% superior.

Therefore, the two key parameters that are indispensable to obtain high SA—and consequently a higher confidence in assigning the correct molecular formula to the compound of interest—are resolution and signal intensity. However, the presence of compounds with close *m/z* values interfering with the abundance of the isotopic pattern cannot be resolved in a QqQMS; by increasing the amount of analyte introduced in the system, the contribution of such isobars can be diminished. For the monitoring of organic synthesis reactions, this approach would only be useful if the reaction by-products had isotopic patterns that do not overlap that of the analyte; otherwise, those by-products will interfere with the measurement of SA and injecting higher amounts of sample will not improve the analyte’s signal. Recent studies have shown that signal intensity is essential for both ∆m and SA. In fact, Croley, et al. [14] affirmed that ion suppression—which is a matrix effect that can significantly decrease the signal of a given compound—is in some cases more important than instrumental resolution and accuracy for the measurement of accurate masses. Additionally, Xu, et al. [15] found that ion abundance was the main factor impacting the accuracy of relative ion abundance—a parameter closely related to SA when studying small molecules of biological interest. Since the QqQMS has low mass resolution compared to QqTOF or Orbitrap mass spectrometers, signal intensity becomes an even more important parameter for the correct assignment of molecular formulas. In a recent study on the application of SA in environmental analysis [10], it was shown that the determination of accurate masses of spiked contaminants in river water extracts using a QqQMS was possible at the highest concentration (300 μg·L^−1^), but not at the lowest concentration tested (80 μg·L^−1^). Therefore, the authors concluded that the low resolution of the QqQMS did not allow the measurement of accurate masses and spectral accuracy of small molecules at trace levels in complex matrices such as river water, but it was possible to perform such analyses in “cleaner” samples with compounds at higher concentrations.

**Figure 4:**
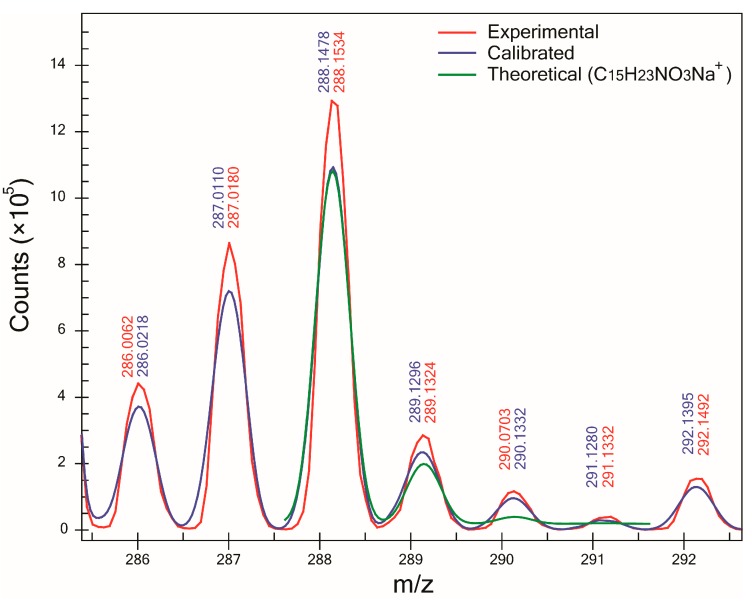
Experimental and calibrated mass spectra and theoretical isotopic pattern of the sodium adduct ion of compound A (C_15_H_23_NO_3_Na^+^). SA was 93.4% and ∆m was −9.2 mDa. While the relative abundance of the isotopic pattern was low, the absence of intense peaks in the profile mass range window (region between −0.5 and 3.5 Da relative to the monoisotopic peak) allowed a good match between calibrated and theoretical isotopic patterns.

Nevertheless, it was observed that for compound A (Figure 4), even if its monoisotopic peak had a low relative abundance (≈3.5%), compared to the base peak, an acceptable SA could be measured (93.4%). This was possible because of the absence of major peaks that could interfere with the isotopic pattern, and peaks M+2 and M+3 were just above the background signal of the instrument. This result suggests that both mass and SA can be measured accurately as long as the profile mass range window of a compound of interest is not affected by the presence of peaks coming from sample contaminants or the matrix and the signal of the isotopic pattern of the compound of interest is above the noise of the instrument.

## 4. Conclusions

A new method for the determination of the mass accuracy (∆m) of small organic molecules using low-resolution mass spectrometry followed by post-acquisition calibration was compared to the traditional method based on high-resolution mass spectrometry. Results showed that with the new method, it was possible to obtain ∆m ≤ 9.2 mDa. While the performance of the traditional method was better (∆m ≤ 2.6 mDa), the new method also measures spectral accuracy (SA), which improves the confidence in the assignment of a molecular formula to a given accurate mass. For the six compounds tested, the correct molecular formula was always ranked among the top seven candidates based on SA. When considering the context of the reaction, the correct formula was the only possible among the potential candidates. It was observed that the presence of peaks coming from sample contaminants in the profile mass range window (−0.5 to 3.5 Da relative to the monoisotopic peak) decreased the quality of both ∆m and SA measurements. Thus, better results can be obtained by infusing higher concentrations of the compounds having low ionization efficiencies or by using liquid chromatography-mass spectrometry to obtain separation of the compounds of interest from sample interferences. These measures should help to reduce the potential contribution of interfering peaks with minimal changes in the proposed method since MassWorks allows post-acquisition calibration using signals with different retention times.

The proposed new method allows for the use of either quadrupole or triple quadrupole mass spectrometers—traditionally used for quantitative analysis—as accurate mass instruments. The implementation of this method demands minimal investment and has the added benefit of ease and robustness of quadruple mass spectrometers compared to more expensive high-resolution mass spectrometers.

## Figures and Tables

**Table 1:** Comparison between the results obtained with the Quattro Premier triple quadrupole mass spectrometer (QqQMS) and the quadrupole-time-of-flight mass spectrometer (QqTOFMS) for the determination of accurate masses of the six compounds analyzed.

			QqQMS before Post-Acquisition Calibration	QqQMS after Post-Acquisition Calibration			QqTOFMS with External Calibration	
Compound	Ion	Exact Mass (*m/z*)	∆m (mDa)	∆m (mDa)	SA (%)	Rank/Possible Formulas	∆m (mDa)	Possible Formulas
A	M+Na^+^	288.1570	−4	−9.2	93.4	5/28	−0.2	20
B	M+H^+^	206.1539	22	−0.1	98.9	1/6	0.3	6
C	M+Na^+^	280.1883	353	1.6	90.6	5/14	−2.6	14
D	M+H^+^	222.1852	50	3.0	95.1	1/7	0.1	6
E	M+Na^+^	308.2196	381	4.6	93.3	7/13	−0.1	14
F	M+Na^+^	201.0289	247	7.3	98.5	3/32	2.5	31

∆m: mass accuracy; SA: spectral accuracy. All samples were infused once, and typically ≈30 acquisitions per sample were recorded for the QqTOFMS measurements and ≈100 acquisitions per sample for the QqQMS.

## Supplementary Materials

The following are available online at http://www.mdpi.com/2409-9279/1/1/9/s1, Table S1: Compound A, Table S2: Compound B, Table S3: Compound C, Table S4: Compound D, Table S5: Compound E, Table S6: Compound F.
