# Supplementary material for: Method for the Routine Determination of Accurate Masses by Triple Quadrupole Mass Spectrometry
Source: Methods Protoc. 2018 Feb 14;1(1):9. doi: 10.3390/mps1010009 (PMC6526401; doi:10.3390/mps1010009)
Supplement: Supplementary file 1 [file mps-01-00009-s001.pdf]

Method for the routine determination of accurate masses by triple quadrupole mass spectrometry

Pedro A. Segura*, Killian Barry, Emmanuel Eysseric, Shawn Gallagher-Duval, Philippe Venne, Guillaume Bélanger

* Tel: 1-(819) 821-7922. Fax: 1-(819) 821-8019. E-mail: pa.segura@usherbrooke.ca

Department of Chemistry, Université de Sherbrooke, Sherbrooke, QC J1K 2R1

Supplementary material

1. ***Possible formulas for each compound (correct formula is highlighted)***

Table S1. Compound A

Exact mass (Da)	Molecular formula (cationized molecule)	Mass accuracy (mDa)	Spectral accuracy (%)	Rank based on spectral accuracy
288.1383	C20H18NO+	9.5	94.2	1
288.1495	C19H18N3+	-1.7	94.0	2
288.1471	C17H19N3Na+	0.7	93.9	3
288.1455	C14H18N5O2+	2.3	93.4	4
288.1570	C15H23NO3Na+	-9.2	93.4	5
288.1567	C13H18N7O+	-8.9	93.1	6
288.1442	C13H22NO6+	3.6	92.6	7
288.1431	C12H19N5O2Na+	4.7	92.3	8
288.1554	C12H22N3O5+	-7.6	92.3	9
288.1543	C11H19N7ONa+	-6.5	92.0	10

Table S2. Compound B

Exact mass (Da)	Molecular formula (cationized molecule)	Mass accuracy (mDa)	Spectral accuracy (%)	Rank based on spectral accuracy
206.1539	C13H20NO+	-0.1	98.9	1
206.1515	C11H21NONa+	2.3	97.4	2
206.1628	C10H21N3Na+	-9.0	97.1	3
206.1499	C8H20N3O3+	3.9	95.3	4
206.1612	C7H20N5O2+	-7.4	94.9	5
206.1472	C4H16N9O+	6.6	93.3	6

Table S3. Compound C

Exact mass (Da)	Molecular formula (cationized molecule)	Mass accuracy (mDa)	Spectral accuracy (%)	Rank based on spectral accuracy
280.1995	C13H27N3O2Na+	-9.6	90.6	1
280.1993	C11H22N9+	-9.4	90.6	2
280.1856	C10H23N7ONa+	4.3	90.6	3
280.1880	C12H22N7O+	1.9	90.6	4
280.1883	C14H27NO3Na+	1.6	90.6	5
280.1969	C9H23N9Na+	-7.0	90.6	6
280.1867	C11H26N3O5+	3.2	90.5	7
280.1979	C10H26N5O4+	-8.0	90.5	8
280.1907	C16H26NO3+	-0.8	90.2	9
280.1840	C7H22N9O3+	5.9	90.1	10

Table S4. Compound D

Exact mass (Da)	Molecular formula (cationized molecule)	Mass accuracy (mDa)	Spectral accuracy (%)	Rank based on spectral accuracy
222.1852	C14H24NO+	3.0	95.1	1
222.1965	C13H24N3+	-8.3	95.1	2
222.1828	C12H25NONa+	5.4	94.8	3
222.1941	C11H25N3Na+	-5.9	94.7	4
222.1812	C9H24N3O3+	7.0	93.6	5
222.1925	C8H24N5O2+	-4.3	93.4	6
222.1785	C5H20N9O+	9.7	92.2	7

Table S5. Compound E

Exact mass (Da)	Molecular formula (cationized molecule)	Mass accuracy (mDa)	Spectral accuracy (%)	Rank based on spectral accuracy
308.2180	C13H30N3O5+	6.2	93.4	1
308.2169	C12H27N7ONa+	7.3	93.4	2
308.2292	C12H30N5O4+	-5.0	93.4	3
308.2282	C11H27N9Na+	-4.0	93.3	4
308.2308	C15H31N3O2Na+	-6.6	93.3	5
308.2306	C13H26N9+	-6.4	93.3	6
308.2196	C16H31NO3Na+	4.6	93.3	7
308.2193	C14H26N7O+	4.9	93.3	8
308.2153	C9H26N9O3+	8.9	92.9	9
308.2333	C17H30N3O2+	-9.1	92.8	10

Table S6. Compound F

Exact mass (Da)	Molecular formula (cationized molecule)	Mass accuracy (mDa)	Spectral accuracy (%)	Rank based on spectral accuracy
201.0286	C5H6N6OCl+	7.6	98.5	1
201.0398	C4H6N8Cl+	-3.6	98.5	2
201.0289	C7H11O3ClNa+	7.3	98.5	3
201.0401	C6H11N2O2ClNa+	-3.9	98.4	4
201.0425	C8H10N2O2Cl+	-6.3	98.2	5
201.0313	C9H10O3Cl+	4.9	97.9	6
201.0262	C3H7N6OClNa+	10.0	97.4	7
201.0374	C2H7N8ClNa+	-1.2	97.1	8
201.0273	C4H10N2O5Cl+	8.9	97.0	9
201.0385	C3H10N4O4Cl+	-2.3	96.8	10
